# Exploring the Chorioallantoic Membrane (CAM) as a Platform for Burn Wound Modelling and Analysis

**DOI:** 10.3390/mps8040079

**Published:** 2025-07-10

**Authors:** Rita Araújo, Maria Guerra-Gomes, Joana Barros, Pedro Gomes

**Affiliations:** 1 BoneLab, Faculdade de Medicina Dentária, Universidade do Porto, Rua Dr. Manuel Pereira da Silva, 4200-393 Porto, Portugal; rita_araujosilva@sapo.pt (R.A.); mguerragomes@gmail.com (M.G.-G.); 2 LAQV/REQUIMTE, Faculdade de Medicina Dentária, Universidade do Porto, Rua Dr. Manuel Pereira da Silva, 4200-393 Porto, Portugal; 3 i3S, Instituto de Investigação e Inovação em Saúde, Universidade do Porto, Rua Alfredo Allen 208, 4200-135 Porto, Portugal; joana.barros@ineb.up.pt; 4 CECAV—Veterinary and Animal Research Centre UTAD, Universidade de Trás-os-Montes e Alto Douro, Quinta de Prados, 5000-801 Vila Real, Portugal; 5 Associate Laboratory for Animal and Veterinary Sciences (AL4AnimalS), Universidade de Trás-os-Montes e Alto Douro, Quinta de Prados, 5000-801 Vila Real, Portugal; 6 INEB, Instituto de Engenharia Biomédica, Universidade do Porto, Rua Alfredo Allen 208, 4200-135 Porto, Portugal

**Keywords:** burn wound, CAM, chorioallantoic membrane, pre-clinical model

## Abstract

Burn wounds present a significant challenge to both the medical and scientific communities, contributing to the global economic burden on healthcare systems. Due to the complexity and highly variability of burn injuries, along with intricate pathophysiological mechanisms, the development of appropriate and effective treatment strategies remains particularly demanding. The development of robust pre-clinical models that recapitulate specific molecular and cellular events underlying burn injury are essential to advance the understanding of associated biological mechanisms and facilitate the screening of innovative therapeutic interventions. While conventional in vivo models can replicate the key aspects of human burn wound pathology, they are often associated with ethical, logistical, and cost-related limitations. In this context, this study aims to explore the potential of the chicken chorioallantoic membrane (CAM) as an alternative model for burn wound research. Thus, we describe a reproducible and ethically favorable protocol for establishing standardized burn injuries on the CAM and provide a comprehensive evaluation of tissue responses through macroscopic, morphometric, and histological analyses. Our findings support the CAM as a viable pre-clinical platform for the study of burn wound healing and for the early-stage screening of candidate therapeutic agents.

## 1. Introduction

Burn wounds are still, today, a significant challenge within healthcare, presenting highly variable etiology and clinical presentation, often associated with unpredictable prognosis [1]. They deeply impact both functionality and esthetics, significantly reducing patients’ quality of life. Moreover, burn wounds greatly contribute to the economic burden on healthcare systems. According to the World Health Organization, over 11 million people sustain burn injuries each year, with more than 180,000 resulting in death [1,2]. Therefore, the development of therapeutic interventions that promote tissue repair and regeneration, mitigating their negative impact, is a pressing concern [2,3]. The skin is the largest organ in humans, which acts as a barrier between the external and internal environments and plays a critical role in the regulation of body temperature, the prevention of water loss, and protection against pathogen invasion, among other essential physiological functions [4].

Skin regeneration following injury is a complex biological process orchestrated by the immune system, which progresses through four overlapping stages. It initiates with the hemostasis phase, in which platelets are activated to form a blood clot. Among all the cellular elements involved, platelets are arguably some of the most important due to the functional roles they play in hemostasis, inflammation, and regeneration. They respond rapidly to vessel injury, regulating angiogenesis and innate immunity [5]. Then, the inflammation phase follows, during which immune cells prevent pathogenic invasion and eliminate tissue debris from the wound. In the proliferation phase, tissues are reorganized through the migration and proliferation of keratinocytes and fibroblasts, alongside angiogenic activation. Lastly, during the remodeling phase, the extracellular matrix undergoes structural reorganization, restoring tissue architectural features. Ultimately, the recovery of lost functionality occurs via re-epithelization and adequate connective tissue remodeling [6,7,8].

Major concerns associated with burn wound healing include excessive scarring—often a result of exacerbated inflammatory process and inadequate angiogenic response [8,9]—and a high risk of wound infections by opportunistic pathogens [9]. According to Singer and Boyce, timely wound closure is the most critical determinant in achieving favorable clinical outcomes, restoring both function and esthetics [3].

In this sense, an effective therapeutic agent for enhanced burn wound healing should not only actively contribute to accelerating epithelial repair but also promote the remodeling of the underlying connective tissues [3,7]. To support the development of such therapies, suitable pre-clinical models that recapitulate the intricate pathophysiology of burn wounds are essential [10]. Undoubtedly, in vivo models remain the most relevant systems for this purpose. The most widely used burn wound models rely on the use of pigs, followed by rodents and rabbits [11]. However, the use of such animal species is associated with several limitations. The pig model has gained increasing relevance due to the close histological, physiological, and immunological resemblance between porcine and human skin. However, pigs are prone to infections at the wound site, which can complicate healing dynamics and interfere with the interpretation of experimental outcomes. Moreover, they require extensive housing conditions and long experimental periods, limiting the practicality of their use—especially for high-throughput or early-stage screening studies [11]. Rodents are more practical for laboratory use due to their low maintenance requirements, short life cycles, and well-characterized genetics. However, rodent skin differs markedly from human skin in several key aspects: it is thinner, is more loosely attached to underlying tissue, and heals primarily through wound contraction rather than re-epithelialization—a process that is not representative of human wound healing [10,11]. Rabbits present an intermediate option, as they are relatively easy to maintain and offer housing advantages over pigs, making them a more practical choice in many laboratory settings. Rabbit skin offers certain advantages over rodent models, such as a greater elasticity and larger surface area, which facilitates experimental manipulation and tissue sampling [12,13].

Moreover, across all these species, burn induction, regardless of the method employed, causes pain and tissue trauma, raising significant ethical considerations [10,11]. As a result, the implantation of strategies aiming to reduce animal suffering are, today, actively preconized by several agencies with the 3Rs framework—Replacement, Reduction, and Refinement in animal use [14]. In this sense, alternative models, aligning with these principles, should be further explored, optimized, and validated.

In this context, the chicken chorioallantoic membrane (CAM) emerges as a promising platform to establish a burn wound model [14]. The CAM is an extra-embryonic tissue that begins to form *in ovo* around day 3 of development (ED3), to sustain embryonic growth via nutrient and gas exchange. It results from the fusion of the chorionic and the allantoic membranes [15,16]. A layer of connective tissue interconnects the two epithelia, harboring a dense and developing vascular network that matures in a predictable pattern until day ten of development (ED10) [15,16]. This particular feature has leveraged the use of CAM for multiple experimental purposes within varied research fields, ranging from its seminal use in irritation assays to cancer biology studies or angiogenesis-related research [17,18]. Importantly, the CAM model offers ethical advantages over traditional animal models. As the central nervous system of the avian embryo remains underdeveloped during the first two-thirds of embryogenesis, it is widely considered to have a limited pain perception during this period [18]. This characteristic aligns with the principles of the 3Rs—particularly Replacement and Refinement—by minimizing animal suffering and offering a viable alternative to higher vertebrate models [19].

Although most CAM-based studies have leveraged its highly vascularized environment—particularly in angiogenesis and tumor research—its application in burn wound modeling remains largely underexplored. This approach requires methodological validation and standardization to establish its reliability in this context [19,20].

Herein, we present a methodological framework for establishing a burn wound model using the CAM, emphasizing its potential as a pre-clinical platform for evaluating the modulatory potential of therapeutic interventions. Additionally, we address critical aspects and common pitfalls of the experimental design to support reproducibility and translational relevance.

## 2. Materials and Methods

### 2.1. Preparation of the CAM

The preparation of the CAM for the *in ovo* model was performed as described previously [21]. Briefly, fertilized chicken eggs (*Gallus domesticus*) were acquired from a local certified vendor (PintoBar, Amares, Braga, Portugal) and incubated horizontally at a temperature of 37 °C with 60% relative humidity, in an Octagon 40 ECO rotary egg incubator (Brinsea, Birmingham, UK). On the 3rd day of development (ED3), the eggs were prepared for CAM access. First, a small puncture was first made at the blunt end of the eggs (air sac) and approximately 4 mL of albumen was aspirated using an 18-gauge needle mounted in a disposable syringe, in order to lower the embryo and facilitate windowing. The puncture site was sealed with a small piece of sterile duct tape to prevent air infiltration. Then, a window was created in the shell at the top of the eggs (blunt pole), to allow direct access to the CAM. Embryo viability was confirmed via the visual assessment of cardiac activity. The window was then covered with sterile duct tape to maintain sterility and prevent dehydration. The eggs were returned to the incubator and maintained under the same conditions until the selected embryonic day for the induction of the burn wound and throughout the experimental period.

### 2.2. Burn Wounds

**Figure 1:**
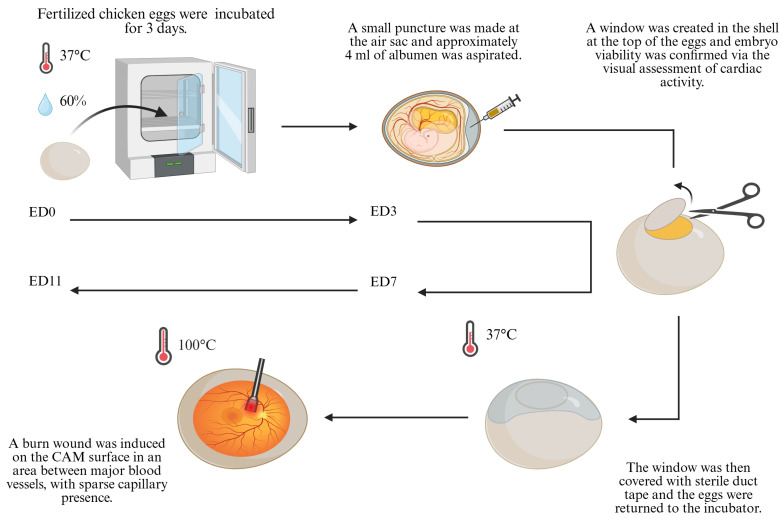
Schematic representation of the experimental workflow.

On the 7th day of embryonic development (ED7), a burn wound was induced on the CAM surface (Figure 1) using a metal stamp with a flat circular tip, preheated to a temperature of 100 °C (see Appendix A, Note 1). A uniform circular burn wound, with a diameter of 4 mm, was created by gently applying the heated stamp to the surface of the CAM, for one second. To ensure experiment reproducibility, the stamp was reheated to the same temperature prior to each burn, allowing it to fully cool between applications.

The location of each burn was carefully chosen in areas of interest between major blood vessels, with sparse capillary presence, to minimize the risk of excessive hemorrhaging or embryo lethality (see Appendix A, Notes 2 and 3). Following the induction of the burn wounds, the embryos were numbered and monitored over the subsequent days. All experimental replicates were processed in parallel under identical conditions to ensure consistency across replicates. A total of ten replicates were used for this experiment.

### 2.3. Macroscopic Analysis of the Wound Progression

CAMs were visualized and photographed over a 96 h period, at 24 h intervals (T0, T24, T48, and T96). Photo documentation was performed using a stereomicroscope Stemi 305 (Zeiss, Oberkochen, Germany) equipped with a commercial camera, AxioCam 208 (Zeiss, Oberkochen, Germany) (see Appendix A, Note 4). All images were analyzed using the software ImageJ (version 1.54 p) concerning previously defined parameters of the diameter and area of each wound. To assess the diameter of each wound, several lines were drawn between two opposing points, upon image calibration, of each burn wound using the “Line tool”, after proper image calibration. For area measurements, the wound perimeter was outlined manually using the “Freehand Selection” tool to obtain the most accurate representation. The mean values of each of these measures were then used for statistical analysis.

### 2.4. Histological Analysis

In the 48 h stage and at the end of the experiment, the chick embryos were euthanized by adding 2 mL of 4% paraformaldehyde (Sigma Aldrich, St. Louis, MO, USA) directly onto the CAM, for 2 h. This procedure simultaneously ensured tissue fixation for subsequent histological analysis. Both the wounded and adjacent non-wounded regions of the CAM were carefully excised and processed to allow for comparative histological evaluation.

Following fixation, tissues were washed in phosphate-buffered saline (PBS), dehydrated through a graded ethanol series (70%, 95%, and 100%), cleared in xylene, and embedded in paraffin wax. The paraffin-embedded tissues were then sectioned at a 5 µm thickness using a rotary microtome, Leica RM2255 (Leica, Wetzlar, Germany), and mounted onto glass slides. The slides were dried at 60 °C for 1 h and subsequently deparaffinized in xylene, followed by rehydration using descending concentrations of ethanol (100%, 95%, 70%), followed by distilled water.

Staining was performed using a standard hematoxylin and eosin (H&E) protocol. Sections were immersed in Harris hematoxylin, rinsed in running tap water, differentiated in 1% hydrochloric acid, and blued in Scott’s tap water substitute. After a second rinse in tap water, slides were counterstained with 1% eosin Y. The stained sections were then dehydrated using ascending ethanol concentrations, cleared in xylene, and coverslipped.

The obtained histological sections of the wound site and the non-injured CAM were photodocumented using the Axiolab5/Axiocam208 (Zeiss, Oberkochen, Germany) imaging system and subjected to further analysis. A semi-quantitative evaluation was performed, independently and double blindly. The following parameters were considered: the morphology of the chorionic and allantoic epithelial layers, morphology of the connective tissue, presence of exudate, and presence of inflammatory cells.

### 2.5. Statistical Analysis

Statistical analysis was performed using Prism 10.3.1 (Dotmatics, Boston, MA, USA). The variables of wound diameter and wound area were compared across experimental timepoints using the one-way ANOVA, followed by Tukey’s post hoc test for multiple comparisons. Results were considered significant at *p* ≤ 0.05.

## 3. Results

### 3.1. Experimental Design

**Figure 2:**
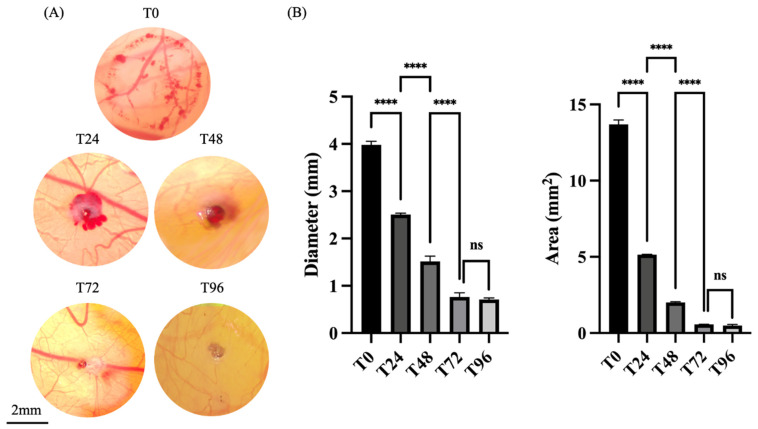
Macroscopic and morphometric analysis of burn wound healing on the CAM. (**A**) Representative macroscopic images of the burn wound at different timepoints, showing the progressive contraction of the lesion over time. (**B**) Quantitative analysis of wound morphometric parameters—wound diameter (mm) and wound area (mm^2^)—highlighting the rapid reduction in the wound size during the initial 72 h post-injury, followed by stabilization. **** *p* < 0.0001; ns-not significant.

Macroscopic observations of the burn wounds immediately after induction showed lesions characterized by a clearly demarcated hyperemic border with minimal hemorrhage (Figure 2A). Morphometric indices calculated at timepoint 0—i.e., wound area and diameter—showed minimal variability across experimental replicates (Figure 2B).

Importantly, the controlled 1 s application of the 4 mm heated metal probe resulted in a localized burn confined to the CAM, without injuring the yolk membrane underneath. This was confirmed by the preservation of an intact sub-CAM vascular network, clearly visible beneath the injury site (Figure 2A). The chosen technique and probe size also effectively prevented excessive bleeding, unwanted CAM adhesion, and tissue charring. The wound placement was consistent and reproducible across the experimental replicates, and the procedures resulted in a 100% embryonic survival rate.

The stage of development of the CAM (ED7) proved optimal, as the placement of the wound between major vessels was achieved with relative ease, minimizing the risk of disrupting the fetal blood supply.

### 3.2. Evolution of the Burn Wound Healing Process

The progression of the burn wound healing in the CAM was evaluated at given timepoints during the 96 h experimental period. A progressive decrease in wound size was observed, indicating active wound contraction and tissue repair over time. Active bleeding had markedly subsided at 24 h, and at the subsequent timepoints, the wound exhibited a hematoma-like appearance that gradually resolved as healing progressed.

Morphometric analysis (Figure 2B) demonstrated that the most significant reductions in wound size occurred between 0 and 72 h. Immediately after burn induction, the wound area reached its maximum, averaging approximately 13.5 mm^2^, reflecting the initial extent of tissue damage. By 24 h, the wound area had decreased by more than half, showing a rapid early healing response. This decreasing trend continued consistently through the 72 h period, by which time the wound diameter had reduced to approximately 1 mm. At 96 h, only minimal additional changes were observed, with no statistically significant differences compared to the 72 h timepoint. This suggests the onset of a plateau phase in the healing process, indicating that most of the tissue contraction and remodeling occurred within the first few days following injury, with later stages characterized by stabilization with limited further reduction (Figure 2B).

### 3.3. Histological Analysis

**Figure 3:**
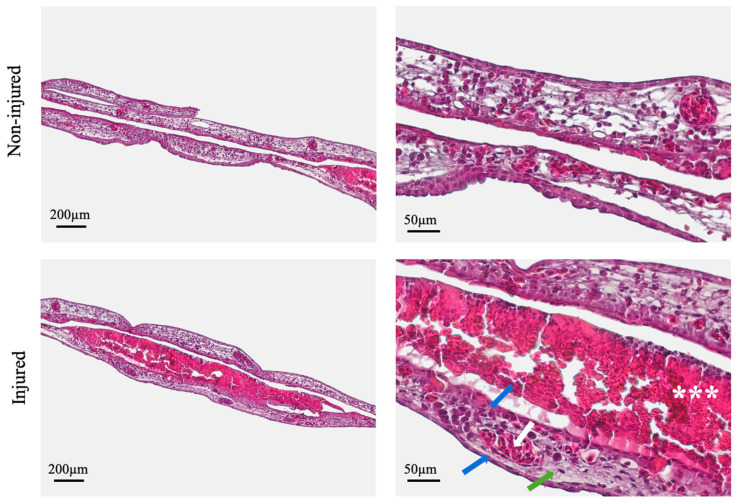
Histological analysis of the CAM. Representative H&E-stained sections of the CAM. The uninjured CAM displays the characteristic trilaminar architecture, with a thin chorionic (ectodermal) epithelium, a vascularized mesodermal connective tissue layer, and an allantoic (endodermal) epithelium. The injured site exhibits comparable histological features, with preserved epithelial integrity and vascularization. Blue arrows: epithelial layers; white arrow: blood vessel containing erythrocytes; green arrow: organized connective tissue; asterisks; granulation tissue and wound exudate. Images from left to right were acquired at 10× and 40× magnification, respectively.

Histological sections from both injured and non-injured regions of the CAM were analyzed at 96 h post-injury (Figure 3). Overall, the CAM maintained its characteristic trilaminar structure, consisting of a loose connective tissue core bordered on both sides by epithelial layers, with interspersed blood vessels of different diameters.

Close observation revealed that the injured regions presented an epithelial architecture closely resembling that of the non-injured areas. Within the regions of healing, no discontinuities, fenestrations, or structural disruptions were present in the chorionic or allontoic epithelial layers. Both epithelia exhibited basophilic nuclei, indicative of active metabolic function consistent with cellular viability (Figure 3, blue arrows). The underlying connective tissue at the injury site displayed a horizontally aligned, coherent fibrillar pattern, similar to that observed in non-injured regions (Figure 3, green arrow). Basophilic fibroblasts were identified at both regions, further supporting tissue viability. No areas of necrosis or tissue destruction were identified. Moreover, blood vessels filled with nucleated erythrocytes were present in both regions, indicating preserved vascular integrity (Figure 3, white arrow). This was accompanied by the presence of several microvessels adjacent to epithelial layers that exhibited no perivascular inflammatory infiltrate and maintained the integrity of the endothelial layer.

A key feature of the injured site was the presence of a central area containing an accumulation of amorphous, eosinophilic material and cellular debris, likely corresponding to wound exudate (Figure 3, white asterisks). This exudate accumulates during the early healing phase and is composed of necrotic tissue remnants, inflammatory infiltrate, and proteinaceous fluid—indicative of early-phase tissue remodeling during the wound healing process.

No evident inflammatory infiltration was noticed within mesodermal connective tissue or in the perivascular areas.

**Figure 4:**
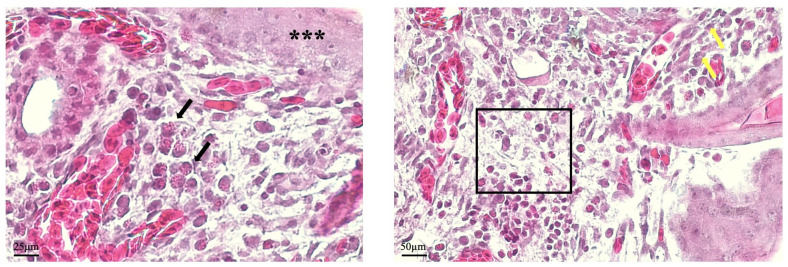
Histological analysis of the CAM after the 48 h experimental period, with representative images illustrating the healing dynamics. The infiltration of avian neutrophils is visualized within the mesodermal connective tissue layer (black arrows). Fibroblasts can be identified within the connective tissue, displaying a disorganized fibrillar matrix (black square). Necrotic epithelial regions are observed (black asterisk), with viable eosinophilic keratinocytes at the basal layer. Keratinocytes are also identified in epithelium neighboring areas (yellow arrows), indicating ongoing tissue modeling.

Specific aspects associated with healing dynamics were assessed in 48 h histological sections. In this stage immune cell infiltration—specifically avian neutrophils—within the mesodermal connective tissue could be noticed (Figure 4, black arrows), identified by the presence of cytoplasmatic granules. The connective tissue exhibited a disorganized fibrillar pattern, although eosinophilic fibroblasts were present. Regarding the epithelium, necrotic areas remained noticeable (Figure 4, black asterisk), despite viable keratinocytes being observed in the basal layer and adjacent epithelial areas (Figure 4, yellow arrows), suggesting the onset of regenerative activity.

## 4. Discussion

The present study provides an in-depth evaluation of an innovative burn wound model established on the chicken CAM, with emphasis on experimental feasibility, biological response, and potential translational relevance. The results converge to demonstrate that the CAM is a suitable alternative platform to establish a pre-clinical burn wound model.

Macroscopic evaluation immediately upon injury demonstrated that the burn wounds could be reliably induced with a heated metal stamp without causing major vascular disruptions or compromising embryo viability due to a lack of adequate blood perfusion—an observation substantiated by the 100% survival rate. Furthermore, the standardized procedure yielded reproducible injuries, as evidenced by consistent wound morphology and dimensions across replicates.

The follow-up observation throughout the experimental period revealed a progressive reduction in the morphometric measurements—wound area and diameter—especially during the first 72 h post-injury. This trend is indicative of the activation of endogenous repair mechanisms facilitating wound contraction and closure. This temporal pattern is a crucial aspect of the model, as it defines the establishment of a responsive window during which potential therapeutic agents may exert modulating effects, offering a meaningful timeframe for pre-clinical testing.

Histological evaluation reinforced macroscopic and morphometric observations. At 96 h post-injury, the CAM at the wound site exhibited continuous epithelial layers, well-aligned connective tissue fibers, viable fibroblasts, and preserved vascular structures—all hallmark features of a coordinated tissue repair response. These findings reflect key biological processes relevant to burn wound healing and validate the CAM’s utility as a model for investigating such responses. Although the CAM differs structurally and physiologically from mammalian skin and mucosa, its reparative response to burn injury effectively recapitulates the two most critical components of wound healing: re-epithelization and connective tissue remodeling [20]. As such, this model provides a promising platform for assessing the efficacy of candidate therapies aimed at accelerating epithelial closure and modulating extracellular matrix dynamics—both crucial for minimizing scarring and promoting functional tissue restoration [3]. The presence of wound exudate composed of inflammatory infiltrate, cellular debris, and protein-rich fluid further supports the model’s capacity to recapitulate acute wound responses, supporting its biological relevance [22,23]. In addition, the experimental protocol offers flexibility to investigate aspects of healing dynamics needed to be explored, as demonstrated by observations of histological sections in the 48 h stage. In this stage, the presence of inflammatory cells can be assessed, combined with the proliferative activity of fibroblasts and keratinocytes. Another relevant aspect of wound healing is the enhanced angiogenesis that sustains granulation tissue formation and ensures adequate nutrient delivery to support the intense cellular activity in this stage [18]. In the CAM, new vessel formation can be assessed, as established previously, by identifying microvessels, which are easily identified in histological samples [18]. Although vascular formation is often assessed in macroscopic images, by counting new vessels arising from the injury area, histological analysis can successfully differentiate angiogenic vessel formation from inflammation-induced neovascularization, the latter typically characterized by perivascular inflammatory infiltrates [16,20].

In this sense, these findings underscore the importance of histological evaluation discerning between true tissue repair and passive wound contraction [20], an aspect not previously explored in the context of CAM-based burn injury models.

A significant advantage of the CAM model lies in its rapid experimental timeline, offering meaningful results within just a few days. This contrasts with conventional in vivo models—such as pigs, which require prolonged experimental periods, increasing both cost and logistical complexity [10,11]. Thus, the CAM model facilitates the high-throughput screening of therapeutic agents, making it an efficient tool for early-phase pre-clinical evaluation. Nonetheless, interspecies comparisons should consider the specific healing dynamics established for each species, since the variability of histophysiological features and healing times limit the direct applicability of findings across models [11].

Regarding our methodology, the results demonstrate that burn wounds can be analyzed in an *in ovo* approach, although the protocol can be easily adapted to an ex ovo experimental approach. Despite improved visualization conditions within ex ovo cultures, this setting compromises nutritive supply to the embryo and requires extremely controlled high-humidity conditions to ensure embryo survival—requirements not necessary in the *in ovo* approach, which offers simpler and less demanding experimental conditions [19]. The attained results also show that the selected wound size (≈13.5 mm^2^) proved appropriate for inducing a subcritical, non-lethal wound, when properly placed, allowing the adequate visual follow-up of wound progression. This aspect facilitates the potential adaptation of the protocol, regarding its timeline, upon the introduction of modulating stimuli.

Of additional relevance is that the use of the CAM aligns with current animal welfare directives that regulate the use of animals in scientific experimentation. Chicken embryos belong to a distinct phylogenetic class from mammals, aligning with the Replacement principle of the 3Rs by offering a non-mammalian alternative. Moreover, prior to day 14 (ED14), the avian central nervous system is not fully developed, significantly minimizing the likelihood of pain perception and thus aligning with the Refinement principle [14,19]. In fact, embryonic day 7 (ED7) was selected for wound induction based on the features of the well-documented CAM development [16,19]. In this stage, the CAM is fully formed upon the fusion of the chorionic and allantoic membranes and remains actively remodeling until day 10 (ED10), which ensures both accessibility and biological responsiveness to alterations to local environmental changes [16,19]. In addition, ED7 offers optimal conditions for the visualization and assessment of wound progression, since the vascular network is still under development [24].

Nonetheless, we recognize some limitations on the use of the CAM model for burn wound healing. While the induced lesions target multiple layers of tissue—similarly to a second-degree burn—they cannot replicate the full complexity of large, chronic, or deep wounds, frequently seen in clinical settings. Also, the narrow experimental window requires strict timepoint monitoring, although this is mitigated by the overall short duration of the model. Still, the model remains highly suitable for the preliminary, high-throughput screening of therapeutic candidates, leveraging the obtention of results, and reducing the reliance on higher vertebrate models in early testing phases.

Despite the fair simplicity of the experimental protocol, we consider that some aspects are fundamental for its successful establishment. These include the proper and precise anatomical placement of the wound to avoid major vessels during burn induction and the rigorous photo documentation of the wound progression—both critical for ensuring consistency and reproducibility.

It is noteworthy that the scope of the present study does not include the use of the CAM model to conduct mechanistic studies. Nonetheless, adaptations can be adapted to meet specific experimental objectives. Molecular biology techniques may be applied to fresh sections of the tissue, prior to fixation. Avian models are limited in the availability of validated antibodies for some techniques as Western blotting; however, chicken embryonic tissues are highly suited for conducting polymerase chain reaction (PCR) and proteomics analysis, which might provide in-depth insight into the mechanistic aspects of burn wound healing [19,25,26].

## 5. Conclusions

In conclusion, the burn wound model developed on the chicken CAM offers a practical, ethically responsible, and biologically relevant platform for the early-stage pre-clinical evaluation of therapeutic interventions. The protocol is technically accessible, reproducible, and aligned with the principles of the 3Rs, making it a valuable alternative to conventional vertebrate models. By addressing key technical and biological parameters—such as wound placement, survival rate, tissue viability, and histological progression—this study establishes a reliable foundation for future research. The CAM model holds significant potential for the high-throughput screening of candidate therapies aimed at enhancing wound healing, reducing scarring, and promoting functional tissue restoration.

## Data Availability

Data are available on request.

## Abbreviations

The following abbreviations are used in this manuscript:

CAM	Chorioallantoic membrane
PBS	Phosphate-buffered saline
H&E	Hematoxylin and eosin
ED	Embryonic day
T0, T24, T48, T72, T96	Experimental timepoints at 0, 24, 48, 72, and 96 h, respectively

## Appendix A. Troubleshooting Notes

**Note 1: Inadequate visualization of CAM anatomy for proper burn wound placement**

The correct placement of the burn wound requires the avoidance of major blood vessels. Therefore, clear visualization is key to selecting the appropriate location. If visibility is limited, the top window can be carefully widened with scissors, taking care to avoid eggshell debris from falling onto the CAM surface. This will expose a larger area and facilitate appropriate site selection.

**Note 2: Excessive bleeding during burn wound induction**

Excessive bleeding upon the experimental procedure may indicate the accidental rupture of a major blood vessel or mechanical tissue tearing. Make sure that the proper visualization of the CAM anatomy is achieved (as described in Note 1). Avoid applying excessive pressure with the heated metal stamp and ensure light, 1 s contact only.

**Note 3: Tissue cauterization or charring following burn wound induction**

Tissue cauterization or charring may result from prolonged contact or the overheating of the metal stamp. Ensure that the metal stamp does not turn flame-red upon heating, which is indicative of excessive temperature. Ensure gentle, brief contact with the CAM, as emphasized in Note 2.

**Note 4: Poor visualization of burn wound during photodocumentation**

Clear imaging of the injury site is key to ensure the accurate morphometric analysis and assessment of the wound progression. Poor visualization may result from excessive light reflection or wound displacement due to CAM growth. Adjust the light sources of the stereomicroscope to minimize glare. If needed, slightly tilt the egg (avoiding excessive tipping) to optimize the angle of view and reduce reflective interference.
